# Exploring the Relation between Contextual Social Determinants of Health and COVID-19 Occurrence and Hospitalization

**DOI:** 10.3390/informatics11010004

**Published:** 2024-01-15

**Authors:** Aokun Chen, Yunpeng Zhao, Yi Zheng, Hui Hu, Xia Hu, Jennifer N. Fishe, William R. Hogan, Elizabeth A. Shenkman, Yi Guo, Jiang Bian

**Affiliations:** 1 Department of Health Outcomes and Biomedical Informatics, University of Florida, Gainesville, FL 32611, USA; 2 Lowe’s Tech Hub, Charlotte, NC 28203, USA; 3 Brigham and Women’s Hospital and Harvard Medical School, Harvard University, Boston, MA 02115, USA; 4 Department of Computer Science, Rice University, Houston, TX 77005, USA; 5 Center for Data Solutions and Department of Emergency Medicine, University of Florida, 655 West 8th Street, Jacksonville, FL 32209, USA

**Keywords:** SARS-CoV-2, coronavirus, real-world data, exposome, incidence

## Abstract

It is prudent to take a unified approach to exploring how contextual social determinants of health (SDoH) relate to COVID-19 occurrence and outcomes. Poor geographically represented data and a small number of contextual SDoH examined in most previous research studies have left a knowledge gap in the relationships between contextual SDoH and COVID-19 outcomes. In this study, we linked 199 contextual SDoH factors covering 11 domains of social and built environments with electronic health records (EHRs) from a large clinical research network (CRN) in the National Patient-Centered Clinical Research Network (PCORnet) to explore the relation between contextual SDoH and COVID-19 occurrence and hospitalization. We identified 15,890 COVID-19 patients and 63,560 matched non-COVID-19 patients in Florida between January 2020 and May 2021. We adopted a two-phase multiple linear regression approach modified from that in the exposome-wide association (ExWAS) study. After removing the highly correlated SDoH variables, 86 contextual SDoH variables were included in the data analysis. Adjusting for race, ethnicity, and comorbidities, we found six contextual SDoH variables (i.e., hospital available beds and utilization, percent of vacant property, number of golf courses, and percent of minority) related to the occurrence of COVID-19, and three variables (i.e., farmers market, low access, and religion) related to the hospitalization of COVID-19. To our best knowledge, this is the first study to explore the relationship between contextual SDoH and COVID-19 occurrence and hospitalization using EHRs in a major PCORnet CRN. As an exploratory study, the causal effect of SDoH on COVID-19 outcomes will be evaluated in future studies.

## 1. Introduction

The COVID-19 pandemic, caused by SARS-CoV-2 infection, has continued for more than 2 years and has had severe health consequences worldwide [1]. This includes dramatic losses of human life and an unprecedented challenge for hospitals and health systems. As of writing, COVID-19 has infected over 772 million people and caused 7 million deaths globally [2]. During the pandemic, COVID-19 forced the hospital and health system to operate with limited capacity and resources, threatening their ability to continue to provide essential services to their patients and communities. According to a Massachusetts state survey, 20% of the respondents are missing either critical urgent care or essential routine care, mostly due to limited health care capacity, while a second review reported a median of a 37% reduction in medical service worldwide [3,4]. The revenue of the hospitals and health systems also decreased by 10% to 80%, limiting their investment in new facilities and technologies [5].

Prior research has shown that a number of social determinants of health (SDoH), especially the social factors (e.g., religious influences, social class) and built environmental factors (e.g., transportation, opportunities for physical activity), are associated with COVID-19 outcomes, including incidence and hospitalization. The WHO defines SDoH as “the conditions in the places where people live, learn, work, and play that affect a wide range of health and quality of life risks and outcomes” [6]. SDoH were proven to have profound effects on life expectancy [7], oral health [8], mental health [9], and the incidence and outcome of diabetes [10,11], cardiovascular disease [12], and various chronic diseases [13]. SDoH can be measured both at individual and contextual levels. Contextual SDoH are the social and built factors within a community or region that influence health outcomes, while individual SDoH are those measured at the patient level [14]. Most of the research on SDoH and COVID-19 has focused on contextual-level factors as they can be retrospectively measured based on patients’ geolocation information. For example, income level and income disparity are reported to be correlated with the incidence of COVID-19 [15–19]. Ethnicity and ethnic disparity are found to be correlated with COVID-19 incidence and mortality in the United States and the United Kingdom [16,20,21]. Occupation is also found as a correlated factor with COVID-19 incidence and mortality [15,22–24]. However, most prior studies examined a limited number of contextual SDoH. These studies may suffer from unmeasured confounding by co-exposures to other unincluded contextual SDoH, especially considering that a number of SDoH are correlated, e.g., the occupation and the income level of the community. Also, many SDoH have not been considered in the previous studies, e.g., walkability, food accessibility and varieties, natural environment (e.g., green space), area deprivation, etc. It would be prudent to make a comprehensive analysis to explore how contextual SDoH impact COVID-19 outcomes, which would support efficient resource planning and the design of interventions that aim to reduce COVID-19 incidence and hospitalization.

A number of COVID-19 research projects are based on EHR data. The topics of these projects, to name a few, include COVID-19 surveillance (data infrastructure and forecasting) [25–28], COVID-19 outcome prediction [29–40], and social determinants of COVID-19 analyses [20,41–43]. In summary, although a number of studies [15–24] have associated contextual SDoH with COVID-19 outcomes, either poor geographical represented data from limited study sites were used or a few contextual SDoH factors were analyzed. There is still a large knowledge gap in our understanding of the relationships between contextual SDoH and COVID-19 outcomes. In this study, multiple contextual SDoH factors were combined with EHR data from a large clinical research network (CRN) in the National Patient-Centered Clinical Research Network (PCORnet) to conduct an exploratory study on the impact of contextual SDoH on COVID-19 occurrence and hospitalization in Florida. Our analysis explored the major contextual SDoH factors that were potentially related to COVID-19 outcomes.

## 2. Materials and Methods

### 2.1. Data Source

We obtained EHR data from OneFlorida+ [44], a large CRN in the national PCORnet funded by the Patient-Centered Outcomes Research Institute (PCORI). OneFlorida+ includes 12 healthcare organizations that provide care through 4100 physicians, 914 clinical practices, and 22 hospitals, covering all 67 Florida counties. OneFlorida+ contains robust longitudinal, linked patient-level real-world data for approximately 15 million patients, including data from EHRs, Medicaid claims, cancer registries, and vital statistics. The OneFlorida+ EHR data are a Health Insurance Portability and Accountability Act (HIPAA) limited data set that contains detailed patient demographic and clinical variables, including demographics, encounters, diagnoses, procedures, vitals, medications, and laboratory results. This study was approved by the University of Florida Institutional Review Board (IRB202001831, date of approval: 1 July 2020).

### 2.2. Study Population

Our study population included a cohort of adult COVID-19 patients and a matching cohort of adult non-COVID-19 patients. We extracted EHR data between 1 January 2020 and 20 May 2021 of patients with valid ZIP codes whose latest address was in Florida. The COVID-19 patients were identified with COVID-19-related diagnosis codes or positive results from COVID-19-related laboratory tests (Supplementary Table S1). The index dates of the COVID-19 patients were defined as the earliest diagnosis of COVID-19. We matched each COVID-19 patient with four randomly selected patients in OneFlorida+ who were not identified as having COVID-19 based on age, sex, and index month. Both COVID-19 patients and non-COVID-19 patients had at least 1 inpatient or 2 outpatient encounters in 2019. This matching ratio was selected based on our previous study [45].

### 2.3. Outcomes

The primary study outcomes were COVID-19 occurrence (having COVID-19 vs. having no COVID-19) and hospitalization (hospitalized vs. outpatient cared COVID-19). Patients with COVID-19 outpatient care were defined as COVID-19 outpatients. Hospitalized COVID-19 patients were those who were hospitalized, admitted to ICU, required mechanical ventilation, or died due to COVID-19. Diagnosis and procedure codes used to identify these COVID-19 outcomes are summarized in Supplementary Table S1.

### 2.4. Exposures

The primary exposures of interest were contextual SDoH (Table 1). Our analysis included 199 variables from 11 factor domains of social and built environments (Supplementary Table S2) including variables on vacant land, social vulnerability, area deprivation, social capital, crime and safety, hospital bed capacity, healthcare status, walkability, food access, food environment, and green space. The vacant land variables were obtained from the US Department of Housing and Urban Development [46]. The social vulnerability was measured with the Social Vulnerability Index obtained from the Centers for Disease Control and Prevention [47]. Area deprivation was measured with the Area Deprivation Index obtained from Neighborhood Atlas [48]. Social capital data were obtained from the US Census Bureau Business Patterns [49], with the types of establishments determined using the North American Industry Classification System (NACIS) codes [50]. Crime and safety variables were obtained from the Uniform Crime Reporting Program [51]. Hospital bed capacity and healthcare status reflected the abundance of the medical resource at the contextual level. Hospital bed capacity variables were obtained from Definitive Healthcare [52]. Healthcare status variables were obtained from the Area Health Resources Files [53]. Walkability measured the ability to access amenities via walking. It was measured using the well-validated walkability index [54]. Food access variables and food environment variables measured the accessibility of and variation in food. The food access and food environment data were obtained from the US Department of Agriculture’s Food Access Research Atlas [55] and Food Environment Atlas [56]. Green space measured the ratio of green space at multiple spatial scales using the normalized difference vegetation index (NDVI) from National Aeronautics and Space Administration based on satellite imaging [57]. All contextual SDoH were spatiotemporally linked to the patients using zip codes. Our linking process followed the method from Hu et al. [58]. For environmental factors measured at spatial scales smaller than ZIP code, e.g., census tract and census block group, the ZIP code level measurement was the average value of the measurements encompassed in the corresponding ZIP code area. For environmental factors measured at spatial scales larger than that of ZIP code, i.e., county, the measurements of the county that contained the ZIP code area were used. The environmental factors for each patient were selected as the most recent available records before their index dates, i.e., the date of earliest evidence of COVID-19 incidence of the COVID-19 patient or the index date of the COVID-19 patient that the non-COVID-19 patient was matched to. Missing data for all contextual SDoH factors were imputed using the chained equation method [59]. The details on the missing imputation were documented in Appendix A.

### 2.5. Covariates

For all patients in the study population, we obtained EHR data on the following variables: age, sex, race, ethnicity, health insurance, and baseline comorbid conditions including atherosclerotic cardiovascular disease (ASCVD), hypertension, diabetes, chronic obstructive pulmonary disease (COPD), cancer, chronic kidney disease, renal disease, myocardial infarction, organ transplant, asthma, cerebrovascular disease, and peripheral vascular disease. Each comorbid condition was identified using diagnosis codes (ICD-9 and ICD-10, Supplementary Table S3) from EHRs that predated 1 January 2020 and coded with a binary variable indicating whether a patient carried the condition or not.

### 2.6. Data Analysis

First, we performed data normalization and standardization on all continuous contextual SDoH variables. Normalization was performed using the bestNormalize R package 1.5.0 [60], which determined the best transformation from a suite of transformations for each variable based on the Pearson P statistics, including the log, square root, exponential, arcsinh, Box–Cox, and Yeo–Johnson transformations. The normalized variables were then standardized into z-scores (mean = 0 and standard deviation = 1). To detect and minimize multicollinearity in the contextual SDoH variables, we calculated the variance inflation factors (VIF) from the regression models fitted with the SDoH variables and removed the variable with the largest VIF iteratively until all the generated VIF valued less than 5. In this process, we removed 113 variables (58 vacant land variables; 26 food access variables; 8 area health resource variables; 6 crime and safety variables; 6 food environment variables; 4 hospital utilization variables; 4 social capital variables; 1 area deprivation index variable). In total, 86 contextual SDoH variables were included in the analysis.

**Figure 1.**
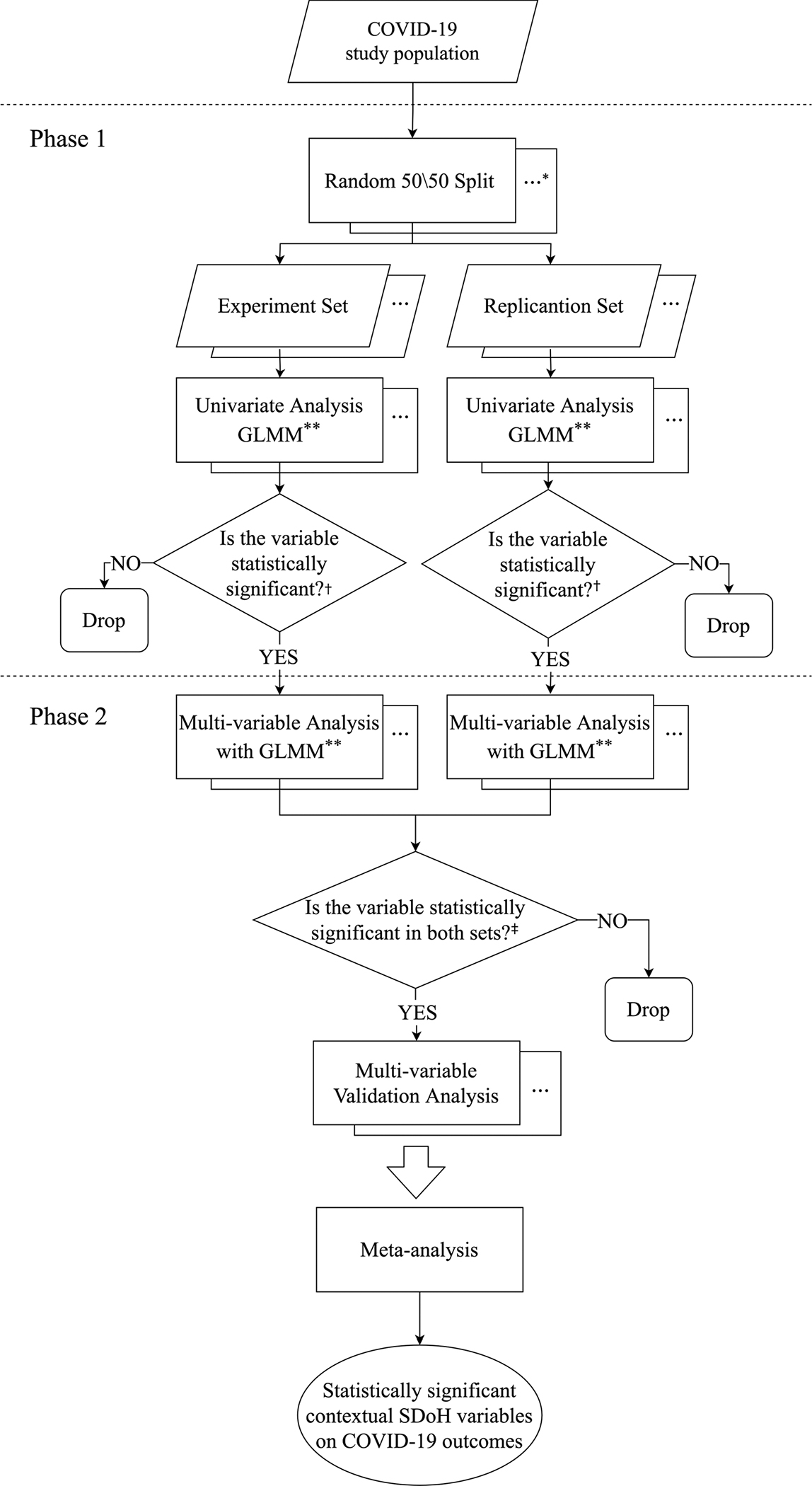
Data analysis flowchart. SDoH: Social Determinants of Health. * Replicated the splits or tests for 100 times; ** GLMM: generalized linear mixed-effect model; ^†^ *p*-value < 4.386 × 10^−4^; ^‡^ *p*-value < 0.05.

Next, we conducted an analysis to examine the associations between the contextual SDoH and two COVID-19 outcomes: (1) having COVID-19 or not, and (2) hospitalized vs. outpatient COVID-19 among COVID-19-positive patients. Our analysis was conducted with a two-phase multiple linear regression approach modified from that in exposome-wide association studies, as shown in Figure 1 [61,62]. Although the original EWAS-MLR method was shown to have inferior performance compared with elastic net, sparse partial least squares regression, graphical unit Evolutionary stochastic search, and the deletion–substitution–addition algorithm, the later methods would not take the geolocation of the patient into consideration. The patients’ geolocation would affect the effect of the SDoH. To control this difference of effect, we adopted GLMM in EWAS-MLR to incorporate the geolocation as the random effect. We first randomly split the dataset into a 50% experiment set and a 50% replication set. The random division was repeated 100 times to create 100 pairs of experiment and replication sets. In Phase 1 of the analysis, we fitted generalized linear mixed-effect models (GLMMs) on the COVID-19 outcomes using each environmental variable alone as the predictor, while adjusting for the covariates in the experiment and replication sets. We used GLMMs to adjust the effects of the SDoH variables for the patients’ geolocation. All 114 contextual SDoH variables were screened in this step and statistically significant variables in either the experiment or replication set were included in the next phase of analysis. To account for multiple testing, we considered a significance threshold of 4.386 × 10^−4^ based on the Bonferroni adjustment [63]. In Phase 2 of the analysis, we fitted GLMMs on the COVID-19 outcomes using contextual SDoH variables found significant in Phase 1 simultaneously as predictors while adjusting for the covariates in the experiment and replication sets. Variables that remained statistically significant in both the experiment and replication sets were then included in the final multivariable models using combined experiment and replication data. A fixed-effect meta-analysis method was then used to generate pooled odds ratios (ORs) and 95% confidence intervals (CIs) for the contextual SDoH variables that were statistically significant based on the 100 final models. All data analyses were performed using Python version 3.8.11.

## 3. Results

### 3.1. Characteristics of COVID-19 and Non-COVID-19 Patients

We summarized the characteristics of our study population in Table 2. Overall, we extracted 15,890 COVID-19 patients and matched them with 63,560 non-COVID-19 patients in OneFlorida+ from January 2020 to May 2021. The COVID-19 and the matched non-COVID-19 patients had comparable distributions for the matching variables of age (mean age was 45.6 and 45.7 years for the COVID-19 and non-COVID-19 patients, respectively [*p* = 0.958]) and sex (sex assigned at birth: percent female was 59.7% and 59.7% in the COVID-19 and non-COVID-19 patients, respectively [*p* = 0.967]). Compared to the non-COVID-19 patients, the COVID-19 patients were more likely to be White (45.1% vs. 34.4%) and Black (28.0% vs. 18.8%), but less likely to be of the Other races (Asian, American Indian or Alaska Native, Native Hawaiian or Other Pacific Islander, or Multiple race: 25.6% vs. 34.5%) (*p* < 0.001). The COVID-19 patients were also more likely to be Hispanic than the non-COVID-19 patients (*p* < 0.001).

Regarding baseline comorbid conditions, the COVID-19 and non-COVID-19 patients had similar percentages of ASCVD (9.3% vs. 9.2%, *p* = 0.753), cancer (4.0% vs. 3.9%, *p* = 0.780), chronic kidney disease (17.7% vs. 17.6%, *p* = 0.587), and myocardial infarction (2.0% vs. 2.1%, *p* = 0.290). The COVID-19 patients were more likely to have had organ transplant (1.8% vs. 0.9%, *p* < 0.001), asthma (0.7% vs. 0.4%, *p* < 0.001), and renal disease (8.8% vs. 8.1%, *p* = 0.003), but less likely to have hypertension (33.9% vs. 36.8%, *p* < 0.001), COPD (12.5% vs. 15.7%, *p* < 0.001), cerebrovascular disease (1.9% vs. 2.4%, *p* < 0.001), and peripheral vascular disease (5.8% vs. 7.3%, *p* < 0.001).

### 3.2. Characteristics of COVID-19 Outpatients and Hospitalized COVID-19 Patients

We summarized the characteristics of the COVID-19 patients by disease severity (outpatient vs. hospitalization) in Table 3. Overall, 78.2% of the COVID-19 patients were outpatient cases, while 21.8% were hospitalized cases. Patients with COVID-19 hospitalization were on average older than COVID-19 outpatients (41.9 vs. 59.4 years, *p* < 0.001). Compared to COVID-19 outpatients, hospitalized COVID-19 patients were more likely to be male (45.5% vs. 38.9%, *p* < 0.001), Black (33.5% vs. 26.4%, *p* < 0.001), and non-Hispanic (68.3% vs. 63.8%, *p* = 0.041). Furthermore, hospitalized COVID-19 patients had significantly higher rates of all baseline comorbid conditions than COVID-19 outpatients.

### 3.3. Associations between Contextual SDoH and COVID-19 Occurrence and Hospitalization

The ORs and 95% CIs of the statistically significant SDoH variables from the Phase 2 analyses of COVID-19 occurrence (having COVID-19 vs. having no COVID-19) are summarized in Table 4. We found six contextual SDoH variables significantly related to the occurrence of COVID-19 adjusted for covariates. Residing in regions with a higher number of pediatric hospital beds (OR: 0.85, 95% CI: 0.85–0.86), higher rate of bed utilization (OR: 0.86, 95% CI: 0.86–0.88), or a higher percentage of vacant business properties (OR: 0.90, 95% CI: 0.89–0.91) was associated with a lower probability of having COVID-19. On the other hand, residing in regions with a higher number of golf courses and country club establishments (OR: 1.05 95% CI: 1.03–1.07), higher murder rate (OR: 1.13, 95% CI: 1.13–1.15), or higher percentage of minorities (OR: 1.30, 95% CI: 1.29–1.31) was associated with a higher probability of having COVID-19.

The ORs and 95% CIs of the statistically significant SDoH variables from the Phase 2 analyses of COVID-19 hospitalization (hospitalized (including ICU care, mechanical ventalized, and demised patient) vs. outpatient care COVID-19) are also summarized in Table 4. Adjusting for the covariates, three contextual SDoH variables were significantly associated with the hospitalization of COVID-19. Residing in regions with a higher percentage of farmers markets that report selling baked/prepared food products (OR: 0.90, 95% CI: 0.86–0.93) was associated with a lower probability of being hospitalized for COVID-19-positive patients. However, residing in regions with a higher percentage of SNAP households (OR: 1.14, 95% CI: 1.06–1.22) or a higher number of establishments in religious organizations (OR: 1.34, 95% CI: 1.16–1.54) was associated with a higher probability of hospitalization for COVID-19 positive patients.

## 4. Discussion

Linking patient data from the OneFlorida+ CRN and contextual SDoH data from various sources, we conducted a study to examine the association between 199 contextual SDoH and COVID-19 occurrence and hospitalization. After removing the highly correlated SDoH variables, a total of 114 contextual SDoH variables were included in our data analysis. Our analysis identified six contextual SDoH variables that were significantly associated with COVID-19 occurrence and three contextual SDoH variables that were significantly associated with COVID-19 hospitalization in COVID-19-positive patients.

Most contextual SDoH found statistically significant with COVID-19 outcomes in our study are consistent with the literature. Our analysis showed that the number of pediatric hospital beds was found related to the COVID-19 incidence. Pediatric hospitals are specialty hospitals that are usually located in large cities with multiple healthcare resources or options and at academic centers where abundant medical resources are available. The number of pediatric hospital beds is thus related to the availability of medical resources, whereas COVID-19 incidence was reported to be lower in affluent communities with abundant medical resources [64]. The number of supplemental nutrition access program (SNAP) households, an indicator of lower socioeconomic status, was also found related to the likelihood of COVID-19 infection and being hospitalized if infected, respectively [15,41]. The number of religious establishments was related to COVID-19 hospitalization. Prior research has shown that religious practices can facilitate the spread of COVID-19 and increase COVID-19 mortality in religious groups [65,66]. An explanation for this observation could be the suboptimal uptake of the COVID-19 vaccine caused by religious factors. A number of studies have reported that religious factors had negative impacts on COVID-19 vaccination in the United States, the United Kingdom, and Africa [67–69]. Research also found religion increases social interactions, besides religious practices, which increases the incidence of COVID-19 [70]. This could indicate that religious practices could be a confounder of COVID-19 outcomes, which warrants further analysis in our future study. Further, consistent with prior research, we found that the murder rate [71–73] and percentage of minorities [15,41,74,75] was related to COVID-19 incidence. However, these relations would not necessarily reflect the causal effect of the SDoH factor on COVID-19 occurrence and hospitalization. Many of these factors were correlated with major determinants, e.g., the percentage of vacant business properties was correlated with the income level of the area, and the number of golf courses and country club establishments were related to the population density. These factors could be the confounder of these major determinants. Also, as we removed the correlated regional income and crime contextual SDoH factors, we could not further identify the effect of the correlated factor on COVID-19 occurrence and hospitalization. Further studies are required to identify the causal effect between the major determinants and COVID-19 occurrence and hospitalization.

We identified some new SDoH factors that are associated with COVID-19 incidence and outcome. First, we found the percentage of vacant business properties was a reflection of the local economic status and was negatively correlated with the area’s prosperity [76]. This would be the result of the low population density in the area as the percentage of vacant business properties was found highly correlated with the percentage of vacant resident properties. Additionally, we also found a higher percentage of farmers markets that report selling baked/prepared food products was associated with a lower likelihood of being hospitalized in COVID-19-positive patients. This factor was not reported before as a factor for COVID-19 incidence or outcome. Upon investigation, we found this factor highly correlated with the percentage of farmers markets that report selling vegetables. This reveals that healthy diet and lifestyle had a great effect on COVID-19 outcomes.

We also identified some relations between contextual SDoH and COVID-19 outcomes that are not intuitive and thus require further investigation. The rate of hospital bed utilization rate was proposed as a measure of a hospital’s ability to function safely and effectively, with high bed utilization being associated with a greater risk of hospital-associated infection [77]. However, we found that higher bed utilization was associated with a lower likelihood of COVID-19 infection. Further analysis revealed a high correlation between hospital bed utilization and the number of available beds, suggesting that bed utilization might be a confounder for the available medical resources. The number of establishments in golf courses and country clubs is an indicator of higher socioeconomic status, whereas we found that it was associated with a higher likelihood of COVID-19 infection, which appears to contradict our findings on the numbers of SNAP households. Future studies are needed to further examine these intuitive relationships.

The main strength of our study is the consideration of multiple contextual SDoH. We were the first to conduct a comprehensive study on the relation between 199 contextual SDoH variables and COVID-19 outcomes. A few limitations need to be noted. First, we were only able to analyze EHR data of COVID-19 patients who resided in Florida. Our findings of the contextual SDoH factors are not necessarily generalizable to the other US regions or states, where state policy and other factors associated with the SDoH differ from that in Florida. Second, due to the observational nature of the study, and the complexity of COVID-19 etiology, our results cannot be used to establish any causal relationship between the contextual SDoH and the COVID-19 outcomes. As an exploratory study, our findings do not suggest causal relations between SDoH factors and COVID-19 incidence or hospitalization. Also, the SDoH factors related to the occurrence and hospitalization of COVID-19 would not be limited to the few identified in our study. As we removed the correlated SDoH factors in our analysis, the effect of these SDoH factors requires more detailed study. Third, there are also many known limitations to EHRs. For example, inaccuracy and vague ICD coding is a known issue to EHRs that could lead to misidentification of patient condition. Also, EHRs do not record death cases outside inpatient cases currently due to not being linked with the government’s death register system. In addition, our experiment assumed independence among the included SDoH factors and comorbidities. Our study method also could not eliminate all the SDoH that correlated with race, ethnicity, and comorbidity. Although the highly correlated SDoH variables were likely to be statistically insignificant [78] in the univariate analysis in our study and had been removed from the multi-variable analysis, this could not guarantee the removal of all correlated SDoH factors. This assumption does not hold in the real-world scenario and we plan to address this issue in our future study to analyze the causal relation between SDoH factors and COVID-19 outcomes.

## 5. Conclusions

This is the first study to examine the relation of contextual SDoH with COVID-19 occurrence and hospitalization using EHRs in a large CRN in the PCORnet. Our study identified nine contextual SDoH variables that related to COVID-19 incidence and hospitalization. Most of these relations aligned with the findings from the previous literature. More in-depth studies are needed to examine the causal relationships between SDoH and COVID-19 outcomes.

## Supplementary Material

The following supporting information can be downloaded at: https://www.mdpi.com/article/10.3390/informatics11010004/s1, Supplementary Table S1: Diagnosis codes for COVID-19 outcomes, Supplementary Table S2: Contextual Social Determinant of Health (SDoH) Variables, Supplementary Table S3: Diagnosis codes for comorbidities.

## Figures and Tables

**Table 1.** Summary of contextual SDoH data sources.

Name	Data Source and Validation Study	Time Range	Spatial Scales	Temporal Scales
Vacant land	Aggregated USPS Administrative Data on Address Vacancies, HUD	2006–2019	Census tract	3-month
Walkability	Walkability Index, the United States Environment Protection Agency	2015	Census block group	Cross-sectional
Food Access	USDA Food Access Research Atlas	2010, 2015 (2011–2014 interpolated)	Census tract	1-year
Food Environment	USDA Food Environment Atlas	2015	County	1-year
Green Space	NASA MODIS	2020	250 m/1 KM	16-day/monthly
CDC Social Vulnerability Index	CDC ATSDR SVI	2000, 2010, 2014, 2016, 2018	County	14–18 month
Area Deprivation Index	Neighborhood Atlas	2013, 2015	County	20 years
Social Capital	United States Census Bureau	1986–2018	Zip-code	1-year
Crime and Safety	Uniform Crime Reporting Program, FBI	Offense:1960–2017 Arrest: 1974–2016	County	1-year
Hospital Utilization	U.S. Department of Health & Human Services	Accessed2020 August	County	Cross-sectional
Healthcare Indicator	Health Resources & Services Administration	2018–2019	County	1-year

USPS: United States Postal Service; USDA: United States Department of Agriculture; NASA: National Aeronautics and Space Administration; MODIS: Moderate Resolution Imaging Spectroradiometer; CDC: Centers for Disease Control and Prevention; ATSDR: Agency for Toxic Substances and Disease Registry; SVI: Social Vulnerability Index; FBI: Federal Bureau of Investigation.

**Table 2.** Patient characteristics in the OneFlorida+ Data Trust 2020–2021 (*n* = 79,450).

	COVID-19 Patients, *n* = 15,890	Non-COVID-19 Patients, *n* = 63,560	*p*-Value

**Age**			
Mean (SD)	45.6 (20.9)	45.7 (20.9)	=0.958

**Sex**			
Female	9483 (59.7%)	37,940 (59.7%)	=0.967
Male	6407 (40.3%)	25,620 (40.3%)	

**Race**			
White	7161 (45.1%)	26,114 (34.4%)	<0.001
Black	4444 (28.0%)	14,235 (18.8%)	
Other	4060 (25.6%)	18,499 (34.5%)	
Unknown	225 (l.4%)	4712(8.8%)	

**Ethnicity**			
Hispanic	5020 (31.6%)	15,665 (24.6%)	<0.001
Non-Hispanic	10,304 (64.8%)	40,650 (64.0%)	
Other	98 (0.6%)	1981 (3.1%)	
Unknown	468 (3.0%)	5264(8.3%)	

**Comorbid Conditions**			
ASCVD	1473 (9.3%)	5840 (9.2%)	=0.753
Hypertension	5388 (33.9%)	23,392 (36.8%)	<0.001
Diabetes	3070 (19.3%)	11,637 (18.3%)	=0.004
COPD	1984 (12.5)	9980 (15.7%)	<0.001
Cancer	630 (4.0%)	2489 (0.39%)	=0.780
Chronic kidney disease	2819 (17.7%)	11,158 (17.6%)	=0.587
Myocardial infarction	310 (2.0%)	1323 (2.1%)	=0.290
Organ transplant	283 (1.8%)	592 (0.9%)	<0.001
Asthma	117 (0.7%)	266 (0.4%)	<0.001
Renal disease	1402 (8.8%)	5132 (8.1%)	=0.003
Cerebrovascular disease	299 (1.9%)	1512 (2.4%)	<0.001
Peripheral vascular disease	923 (5.8%)	4608 (7.3%)	<0.001

SD: standard variation; ASCVD: atherosclerotic cardiovascular disease; COPD: chronic obstructive pulmonary disease.

**Table 3.** COVID-19 patients by severity in the OneFlorida+ Data Trust 2020–2021(*n* = 15,890).

	COVID-19 Outpatients ^a^, *n* = 12,438 (78.2%)	COVID-19 Hospitalized Patients ^b^, *n* = 3452 (21.8%)	*p*-Value

**Age**			
Mean (SD)	41.9 (19.5)	59.4 (19.8)	<0.001

**Sex**			
Female	7603 (61.1%)	1880 (54.5%)	<0.001
Male	4855 (38.9%)	1572 (45.5%)	

**Race**			
White	5504 (44.3%)	1657 (48.0%)	<0.001
Black	3289 (26.4%)	1155 (33.5%)	
Other	3440 (27.6%)	619 (17.9%)	
Unknown	205 (1.6%)	21 (0.6%)	

**Ethnicity**			
Hispanic	4019 (32.3%)	1001 (29.3%)	=0.041
Non-Hispanic	7938 (63.8%)	2366 (68.3%)	
Other	74 (0.6%)	24 (0.7%)	
Unknown	407 (3.3%)	61 (1.7%)	

**Comorbid Conditions**			
ASCVD	708 (5.7%)	765 (22.2%)	<0.001
Hypertension	3165 (25.4%)	2223 (64.4%)	<0.001
Diabetes	1619 (13.0%)	1451 (42.0%)	<0.001
COPD	1240 (10.0%)	744 (21.5%)	<0.001
Cancer	372 (3.0%)	258 (7.5%)	<0.001
Chronic kidney disease	1487 (12.0%)	1332 (38.6%)	<0.001
Myocardial infarction	133 (1.1%)	177 (5.1%)	<0.001
Organ transplant	176 (1.4%)	107 (3.1%)	<0.001
Asthma	76 (0.6%)	41 (1.2%)	<0.001
Renal disease	641 (5.2%)	761 (22.1%)	<0.001
Cerebrovascular disease	147 (1.2%)	152 (4.4%)	<0.001
Peripheral vascular disease	478 (3.8%)	445 (12.9%)	<0.001

SD: standard variation; ASCVD: atherosclerotic cardiovascular disease; COPD: chronic obstructive pulmonary disease.

a Outpatient cases were COVID-19 patients that only had outpatient encounters.

b Hospitalized cases included those who were hospitalized, who were admitted to intensive care units, who used mechanical ventilators, or those who died.

**Table 4.** Associations between contextual SDoH and COVID-19 outcomes in the OneFlorida+ Data Trust 2020–2021.

Contextual SDoH	OR (95% CI)	*p*-Value

***Outcome 1: having COVID-19* vs. *having no COVID-19***		
Number of pediatric hospital beds (pediatric beds)	0.86 (0.85, 0.86)	<0.001
Rate of hospital bed utilization (hospital bed utilization)	0.87 (0.86, 0.88)	<0.001
Percentage of vacant business properties (12 to 24 months) (vacant property)	0.90 (0.89, 0.91)	<0.001
Number of establishments in golf courses and country clubs (per 10,000 population) (number of golf courses)	1.05 (1.03, 1.07)	<0.001
Murder rate (per 100 population)	1.14 (1.13, 1.15)	<0.001
Percentage of minority (all persons except white, non-Hispanic) (percent of minority)	1.30 (1.29, 1.31)	<0.001

***Outcome 2: hospitalized* vs. *outpatient cared COVID-19***		
Percentage of farmers markets that report selling baked/prepared food products (farmers market)	0.90 (0.86, 0.93)	<0.001
SNAP households, low access to stores (low access)	1.14 (1.06, 1.22)	<0.001
Number of establishments in religious organizations (per 10,000 population) (religion)	1.34 (1.16, 1.54)	<0.001

## Data Availability

The OneFlorida+ electronic health record data are considered Protected Health Information under the Health Insurance Portability and Accountability Act of 1996 (HIPAA) in the United States and as such are not publicly available. The vacant land information is available from the Aggregated USPS Administrative Data on Address Vacancies at https://www.huduser.gov/portal/datasets/usps.html (accessed on 2 October 2020). The walkability index is available from the United States Environment Protection Agency at https://edg.epa.gov/metadata/catalog/search/resource/details.page?uuid=%7B251AFDD9-23A7-4068-9B27-A3048A7E6012%7D (accessed on 2 October 2020). The food access measurement is available from the United States Department of Agriculture Food Access Atlas at https://www.ers.usda.gov/data-products/food-accessresearch-atlas/ (accessed on 2 October 2020). The food environment measurements are available from the United States Department of Agriculture Food Environment Atlas at https://www.ers.usda.gov/data-products/food-environment-atlas/ (accessed on 2 October 2020). The green space measurements are available from the National Aeronautics and Space Administration Moderate Resolution Imaging Spectroradiometer program at https://modis.gsfc.nasa.gov/data/ (accessed on 2 October 2020). The CDC social vulnerability index is available from the CDC at https://www.atsdr.cdc.gov/placeandhealth/svi/index.html (accessed on 2 October 2020). The social capital measurements are available from the US Census Bureau at https://www.census.gov/ (accessed 2 October 2020). The crime and safety measurements are available from the Uniform Crime Reporting Program at https://www.fbi.gov/services/cjis/ucr (accessed on 2 October 2020). The hospital utilization measurements are available from the U.S. Department of Health & Human Services at https://protect-public.hhs.gov/pages/hospital-utilization (accessed on 2 October 2020). The healthcare indicators are available from the Health Resources & Services Administration at https://data.hrsa.gov/ (accessed on 2 October 2020).

## Appendix A. Missing Imputation in Contextual SDoH Factors and Covariates

We imputed missing data for all contextual SDoH factors and covariates using the chained equations method with R mice package. We considered variables as predictors in the imputation model if they fulfill the following two criteria. First, the potential predictor variable has more than 40% non-missing values compared to the missing variable. Second, the potential predictor variable was correlated with the missing variable or its missing probability. We allowed the number of predictors in the imputation model to be 25 at maximum. Our data set contained small fractions of missing values and the imputation produced limited impact.
