# Supplementary material for: Exploring the Relation between Contextual Social Determinants of Health and COVID-19 Occurrence and Hospitalization
Source: Informatics (MDPI). Author manuscript; Available in PMC 2025 Aug 6. (PMC12327543; doi:10.3390/informatics11010004)
Supplement: The following supporting information can be downloaded at: https://www.mdpi.com/article/10.3390/informatics11010004/s1, Supplementary Table S1: Diagnosis codes for COVID-19 outcomes, Supplementary Table S2: Contextual Social Determinant of Health (SDoH) Variables, Supplementary Table S3: Diagnosis c [file NIHMS2041663-supplement-The_following_supporting_information_can_be_downloaded_at__https___www_mdpi_com_article_10_3390_informatics11010004_s1__Supplementary_Table_S1__Diagnosis_codes_for_COVID-19_outcomes__Supplementary_Table_S2__Contextual_.pdf]

**Supplemental material**

**Supplement Table S1.** Diagnosis codes for COVID-19 outcomes.

| Outcomes | ICD10/ICD9/CPT4/HC/ HCPCS | LONIC |
|---|---|---|
| **COVID-19 Incidence** | ICD10: B97.29, U07.1, B34.2, B97.2, B97.21, J12.81, U04, U04.9, U07.2 | 94509-7, 94758-0, 94765-5, 94315-9, 94510-5, 94311-8, 94312-6, 94533-7, 94766-3, 94316-7, 94307-6, 94308-4, 94511-3, 94819-0, 94759-8, 94500-6, 94845-5, 94822-4, 94309-2, 94531-1, 94306-8, 94534-5, 94314-2, 94767-1, 94763-0, 94764-8, 94313-4, 94310-0, 94502-2, 94532-9, 94565-9, 95209-3, 94760-6, 94640-0, 94660-8, 94645-9, 94639-2, 94641-8, 94643-4, 94647-5, 94558-4, 95406-5, 95409-9, 94745-7, 94756-4, 94757-2, 94646-7, 95424-8, 95425-5, 95423-0, 94746-5, 94768-9, 94507-1, 94508-9, 94762-2, 94769-7, 94562-6, 94720-0, 94563-4, 94505-5, 94564-2, 94506-3, 95125-1, 94547-7, 94661-6, 94761-4, 95416-4 |
| **Mechanical Ventilation** | PXCH: 31502, 31600, 31601, 31603, 31605, 31610, 31611, 31612, 31613, 31614, 94002, 94003, 94004, 94656, 94657, 4168F, K0165; PX09: 31.2, 31.21, 31.29, 96.70, 96.71, 96.72, 96.04, 96.05, V44.0; PX10: 5A1935Z, 5A1945Z, 5A1955Z, 0BH17EZ, 0BH18EZ | |
| **Admission to ICU** | CPT: 99291, 99292 | |

ICU: Intense Care Unit.

**Supplement Table S2.** Contextual Social Determinant of Health (SDoH) Variables.

| **Contextual SDoH Variables** |
|---|
| **CDC Social Vulnerability Index** |
| Percentage of persons aged 65 and older estimate |
| Percentage of persons aged 17 and younger estimate |
| Percentage of civilian noninstitutionalized population with disability estimated |
| Percentage of single-parent households with children under 18 estimated |
| Percentage minority (all persons except white, non-Hispanic) estimated |
| Percentage of persons (age 5+) who speak English "less than well" estimated |
| Percentage of housing in structures with 10 or more units estimated |
| Percentage of mobile homes estimated |
| Percentage of occupied housing units with more people than rooms estimated |
| Percentage of households with no vehicle available estimated |
| Percentage of persons in institutionalized group quarters estimated |
| Percentage of persons with no high school diploma (25+) estimated |
| Percentage of persons below poverty estimated |
| Unemployment rate estimate |
| Per capita income estimate |
| **Social Capital** |
| ADI state rank |
| **Hospital Utilization** |
| Number of adult ICU beds |
| Average machine ventilator usage |
| Bed utilization |
| Number of ICU beds |
| Number of licensed beds |
| Number of staffed beds |
| Number of pediatric hospital beds |
| Potential increase in bed capacity |
| **Green Space** |
| Normalized difference vegetation index |
| **Walkability** |
| National walkability index |
| **Crime and Safety** |
| Total crime rate (per 100 population) |
| Robbery rate (per 100 population) |
| Motor vehicle theft rate (per 100 population) |
| Murder rate (per 100 population) |
| Larceny rate (per 100 population) |
| Forcible sex offenses rate (per 100 population) |
| Burglary rate (per 100 population) |
| Aggravated assault rate (per 100 population) |
| **Social Capital** |
| Average days addresses vacant |
| Average days addresses no-stat |
| Percentage of vacant addresses |
| Percentage of vacant addresses 3 months to less |

Percentage of vacant addresses 3 months to 6 months
Percentage of vacant addresses 6 months to 12 months
Percentage of vacant addresses 12 months to 24 months
Percentage of vacant addresses 24 months to 36 months
Percentage of vacant addresses 36 months or longer
Percentage of previous quarter vacant addresses currently no-stat
Percentage of no-stat addresses
Percentage of no-stat addresses 3 months to less
Percentage of no-stat addresses 3 months to 6 months
Percentage of no-stat addresses 6 months to 12 months
Percentage of no-stat addresses 12 months to 24 months
Percentage of no-stat addresses 24 months to 36 months
Percentage of no-stat addresses 36 months or longer
Percentage of previous quarter no-stat currently in service
Average days residential addresses vacant
Average days business addresses vacant
Average days residential addresses no-stat
Average days business addresses no-stat
Percentage of residential addresses
Percentage of business addresses
Percentage of other addresses
Percentage of vacant residential addresses among all addresses
Percentage of vacant business addresses among all addresses
Percentage of vacant other addresses among all addresses
Percentage of vacant addresses among residential addresses
Percentage of vacant addresses among business addresses
Percentage of vacant addresses among other addresses
Percentage of vacant residential addresses 3 months to less among all addresses
Percentage of vacant business addresses 3 months to less among all addresses
Percentage of vacant addresses 3 months to less among residential addresses
Percentage of vacant addresses 3 months to less among business addresses
Percentage of vacant residential addresses 3 months to 6 months among all addresses
Percentage of vacant business addresses 3 months to 6 months among all addresses
Percentage of vacant addresses 3 months to 6 months among residential addresses
Percentage of vacant addresses 3 months to 6 months among business addresses
Percentage of vacant residential addresses 6 months to 12 months among all addresses
Percentage of vacant business addresses 6 months to 12 months among all addresses
Percentage of vacant addresses 6 months to 12 months among residential addresses
Percentage of vacant addresses 6 months to 12 months among business addresses
Percentage of vacant residential addresses 12 months to 24 months among all addresses
Percentage of vacant business addresses 12 months to 24 months among all addresses
Percentage of vacant other addresses 12 months to 24 months among all addresses
Percentage of vacant addresses 12 months to 24 months among residential addresses
Percentage of vacant addresses 12 months to 24 months among business addresses
Percentage of vacant addresses 12 months to 24 months among other addresses
Percentage of vacant residential addresses 24 months to 36 months among all addresses
Percentage of vacant business addresses 24 months to 36 months among all addresses
Percentage of vacant addresses 24 months to 36 months among residential addresses

Percentage of vacant addresses 24 months to 36 months among business addresses
Percentage of vacant residential addresses 36 months or longer among all addresses
Percentage of vacant business addresses 36 months or longer among all addresses
Percentage of vacant addresses 36 months or longer among residential addresses
Percentage of vacant addresses 36 months or longer among business addresses
Percentage of previous quarter vacant residential addresses currently in service among all addresses
Percentage of previous quarter vacant business addresses currently in service among all addresses
Percentage of previous quarter vacant addresses currently in service among residential addresses
Percentage of previous quarter vacant addresses currently in service among business addresses
Percentage of no-stat residential addresses among all addresses
Percentage of no-stat business addresses among all addresses
Percentage of no-stat addresses among residential addresses
Percentage of no-stat addresses among business addresses
Percentage of no-stat residential addresses 3 months to less among all addresses
Percentage of no-stat business addresses 3 months to less among all addresses
Percentage of no-stat addresses 3 months to less among residential addresses
Percentage of no-stat addresses 3 months to less among business addresses
Percentage of no-stat residential addresses 3 months to 6 months among all addresses
Percentage of no-stat business addresses 3 months to 6 months among all addresses
Percentage of no-stat addresses 3 months to 6 months among residential addresses
Percentage of no-stat addresses 3 months to 6 months among business addresses
Percentage of no-stat residential addresses 6 months to 12 months among all addresses
Percentage of no-stat business addresses 6 months to 12 months among all addresses
Percentage of no-stat addresses 6 months to 12 months among residential addresses
Percentage of no-stat addresses 6 months to 12 months among business addresses
Percentage of no-stat residential addresses 12 months to 24 months among all addresses
Percentage of no-stat business addresses 12 months to 24 months among all addresses
Percentage of no-stat addresses 12 months to 24 months among residential addresses
Percentage of no-stat addresses 12 months to 24 months among business addresses
Percentage of no-stat residential addresses 24 months to 36 months among all addresses
Percentage of no-stat business addresses 24 months to 36 months among all addresses
Percentage of no-stat addresses 24 months to 36 months among residential addresses
Percentage of no-stat addresses 24 months to 36 months among business addresses
Percentage of no-stat residential addresses 36 months or longer among all addresses
Percentage of no-stat business addresses 36 months or longer among all addresses
Percentage of no-stat addresses 36 months or longer among residential addresses
Percentage of no-stat addresses 36 months or longer among business addresses
Percentage of previous quarter no-stat residential addresses currently in service among all addresses
Percentage of previous quarter no-stat business addresses currently in service among all addresses
Percentage of previous quarter no-stat addresses currently in service among residential addresses
Percentage of previous quarter no-stat addresses currently in service among business addresses
Average days other addresses vacant

**Food Access**

Percentage of SNAP households, low access to stores, 2015
Percentage of farms with direct sales, 2012
Percentage of farms with sales, 2012

**Food Environment**

Percentage of Farmers' markets that report accepting snap, 2018
Percentage of Farmers' markets that report accepting WIC, 2018
Percentage of Farmers' markets that report accepting WIC cash, 2018
Percentage of Farmers' markets that report accepting SFMNP, 2018
Percentage of Farmers' markets that report accepting credit cards, 2018
Percentage of Farmers' markets that report selling fruit & vegetables, 2018
Percentage of Farmers' markets that report selling animal products, 2018
Percentage of Farmers' markets that report selling baked/prepared food products, 2018
Percentage of Farmers' markets that report selling other food products, 2018

**Area Deprivation Index**

Adult diabetes rate, 2013
Flag for low-income low access tracts at 0.5 miles for urban and 10 miles for rural
Flag for low-income low access tracts at 1 mile for urban and 20 miles for rural
Flag for low-income low access tracts at 1 mile for urban and 10 miles for rural
Flag for low access tract using vehicle access and at 20 miles in rural areas
Flag for low access tract at 20 miles
Flag for low access tract at 1 mile
Flag for low access tract at 10 miles
Flag for low access tract at 0.5 mile
Percentage of low access population that are seniors at 0.5 mile
Percentage of low access population that are seniors at 20 miles
Percentage of low access population that are seniors at 1 mile
Percentage of low access population that are seniors at 10 miles
Percentage of seniors age 65+ with low access at 1 mile for urban and 10 miles for rural
Percentage of low access population at 0.5 mile
Percentage of low access population at 20 miles
Percentage of low access population at 1 mile
Percentage of low access population at 10 miles
Percentage of low access population at 1 mile for urban and 20 miles for rural
Percentage of low access population at 1 mile for urban and 10 miles for rural
Percentage of low access population at 0.5 miles for urban and 10 miles for rural
Percentage of low access population that is low income at 0.5 mile
Percentage of low access population that is low income at 20 miles
Percentage of low access population that is low income at 1 mile
Percentage of low access population that is low income at 10 miles
Percentage of low income and low access population at 1 mile for urban and 20 miles for rural
Percentage of low access population at 1 mile for urban and 10 miles for rural
Percentage of low income and low access population at 0.5 miles for urban and 10 miles for rural
Percentage of low access population that are kids at 0.5 mile
Percentage of low access population that are kids at 20 miles
Percentage of low access population that are kids at 1 mile
Percentage of low access population that are kids at 10 miles
Percentage of children aged 0-17 with low access at 1 mile for urban and 10 miles for rural
Percentage of low access population with housing units without vehicle access at 0.5 mile
Percentage of low access population with housing units without vehicle access at 20 miles
Percentage of low access population with housing units without vehicle access at 1 mile
Percentage of low access population with housing units without vehicle access at 10 miles

| |
|---|
| Percentage of low access population with housing units without vehicle access at 1 mile for urban and 10 miles for rural |
| Flag for low access tract at 0.5 miles for urban and 10 miles for rural |
| Flag for low access tract at 1 mile for urban and 20 miles for rural |
| Flag for low access tract at 1 mile for urban areas and 10 miles for rural areas |
| Number of establishments in sports teams and clubs per 10000 population |
| Number of establishments in religious organizations per 10000 population |
| Number of establishments in fitness and recreational sports centers per 10000 population |
| Number of establishments in professional organizations per 10000 population |
| Number of establishments in political organizations per 10000 population |
| Number of establishments in labor organization per 10000 population |
| Number of establishments in golf courses and country clubs per 10000 population |
| Number of establishments in civic and social associations per 10000 population |
| Number of establishments in business associations per 10000 population |
| Number of establishments in bowling center per 10000 population |
| Mortality per 100,000 people |
| Number of good air quality days in 2018 |
| Median household income |
| Median age |
| Average household size |
| Population density per square mile |
| The density of community health facilities per 10000 population |
| The density of primary care physicians per 10000 population |
| The density of nonphysicians per 10000 population |

SDoH: Social Determinants of Health; ADI: Area Deprivation Index; ICU: Intense Care Unit; SNAP: Supplemental Nutrition Access Program; WIC: Special Supplemental Nutrition Program for Women, Infants, and Children; SFMNP: Seniors Farmers' Market Nutrition Program.

**Supplement Table S3.** Diagnosis codes for comorbidities.

| Comorbidity | ICD10/CPT4/HCPCS | ICD9/CPT4/HCPCS |
|---|---|---|
| **ASCVD** | I25.10, I25.11 | 414.01, 429.2 |
| **Hypertension** | H35.031, H35.032, H35.033, H35.039, I10, I11.0, I11.9, I12.0, I12.9, I13.0, I13.10, I13.11, I13.2, I15.0, I15.1, I15.2, I15.8, I15.9, I67.4, N26.2 | 362.11, 401.0, 401.1, 401.9, 402.00, 402.01, 402.10, 402.11, 402.90, 402.91, 403.00, 403.01, 403.10, 403.11, 403.90, 403.91, 404.00, 404.01, 404.02, 404.03, 404.10, 404.11, 404.12, 404.13, 404.90, 404.91, 404.92, 404.93, 405.01, 405.09, 405.11, 405.19, 405.91, 405.99, 437.2 |
| **Diabetes** | E08.00, E08.01, E08.10, E08.11, E08.21, E08.22, E08.29, E08.311, E08.319, E08.321, E08.3211, E08.3212, E08.3213, E08.3219, E08.329, E08.3291, E08.3292, E08.3293, E08.3299, E08.331, E08.3311, E08.3312, E08.3313, E08.3319, E08.339, E08.3391, E08.3392, E08.3393, E08.3399, E08.341, E08.3411, E08.3412, E08.3413, E08.3419, E08.349, E08.3491, E08.3492, E08.3493, E08.3499, E08.351, E08.3511, E08.3512, E08.3513, E08.3519, E08.3521, E08.3522, E08.3523, E08.3529, E08.3531, E08.3532, E08.3533, E08.3539, E08.3541, E08.3542, E08.3543, E08.3549, E08.3551, E08.3552, E08.3553, E08.3559, E08.359, E08.3591, E08.3592, E08.3593, E08.3599, E08.36, E08.37X1, E08.37X2, E08.37X3, E08.37X9, E08.39, E08.40, E08.41, E08.42, E08.43, E08.44, E08.49, E08.51, E08.52, E08.59, E08.610, E08.618, E08.620, E08.621, E08.622, E08.628, E08.630, E08.638, E08.641, E08.649, E08.65, E08.69, E08.8, E08.9, E09.00, E09.01, E09.10, E09.11, E09.21, E09.22, E09.29, E09.311, E09.319, E09.321, E09.3211, E09.3212, E09.3213, E09.3219, E09.329, E09.3291, E09.3292, E09.3293, E09.3299, E09.331, E09.3311, E09.3312, E09.3313, E09.3319, E09.339, E09.3391, E09.3392, E09.3393, E09.3399, E09.341, E09.3411, E09.3412, E09.3413, E09.3419, E09.349, E09.3491, E09.3492, E09.3493, E09.3499, E09.351, E09.3511, E09.3512, E09.3513, E09.3519, E09.3521, E09.3522, E09.3523, E09.3529, E09.3531, E09.3532, E09.3533, E09.3539, E09.3541, E09.3542, E09.3543, E09.3549, E09.3551, E09.3552, E09.3553, E09.3559, E09.359, E09.3591, E09.3592, E09.3593, E09.3599, E09.36, E09.37X1, E09.37X2, E09.37X3, E09.37X9, E09.39, E09.40, E09.41, E09.42, E09.43, E09.44, E09.49, E09.51, E09.52, E09.59, E09.610, E09.618, E09.620, E09.621, E09.622, E09.628, E09.630, E09.638, E09.641, E09.649, E09.65, E09.69, E09.8, E09.9, E10.10, E10.11, E10.21, E10.22, E10.29, E10.311, E10.319, E10.321, E10.3211, E10.3212, E10.3213, E10.3219, E10.329, E10.3291, E10.3292, E10.3293, E10.3299, E10.331, E10.3311, E10.3312, E10.3313, E10.3319, E10.339, E10.3391, E10.3392, E10.3393, E10.3399, E10.341, E10.3411, E10.3412, E10.3413, | 249.00, 249.01, 249.10, 249.11, 249.20, 249.21, 249.30, 249.31, 249.40, 249.41, 249.50, 249.51, 249.60, 249.61, 249.70, 249.71, 249.80, 249.81, 249.90, 249.91, 250.00, 250.01, 250.02, 250.03, 250.10, 250.11, 250.12, 250.13, 250.20, 250.21, 250.22, 250.23, 250.30, 250.31, 250.32, 250.33, 250.40, 250.41, 250.42, 250.43, 250.50, 250.51, 250.52, 250.53, 250.60, 250.61, 250.62, 250.63, 250.70, 250.71, 250.72, 250.73, 250.80, 250.81, 250.82, 250.83, 250.90, 250.91, 250.92, 250.93, 357.2, 362.01, 362.02, 362.03, 362.04, 362.05, 362.06, 366.41 |

| | | |
|---|---|---|
| | E10.3419, E10.349, E10.3491, E10.3492, E10.3493, E10.3499, E10.351, E10.3511, E10.3512, E10.3513, E10.3519, E10.359, E10.36, E10.37X1, E10.37X2, E10.37X3, E10.37X9, E10.39, E10.40, E10.41, E10.42, E10.43, E10.44, E10.49, E10.51, E10.52, E10.59, E10.610, E10.618, E10.620, E10.621, E10.622, E10.628, E10.630, E10.638, E10.641, E10.649, E10.65, E10.69, E10.8, E10.9, E11.00, E11.01, E11.10, E11.11, E11.21, E11.22, E11.29, E11.311, E11.319, E11.321, E11.3211, E11.3212, E11.3213, E11.3219, E11.329, E11.3291, E11.3292, E11.3293, E11.3299, E11.331, E11.3311, E11.3312, E11.3313, E11.3319, E11.339, E11.3391, E11.3392, E11.3393, E11.3399, E11.341, E11.3411, E11.3412, E11.3413, E11.3419, E11.349, E11.3491, E11.3492, E11.3493, E11.3499, E11.351, E11.3511, E11.3512, E11.3513, E11.3519, E11.3521, E11.3522, E11.3523, E11.3529, E11.3531, E11.3532, E11.3533, E11.3539, E11.3541, E11.3542, E11.3543, E11.3549, E11.3551, E11.3552, E11.3553, E11.3559, E11.359, E11.3591, E11.3592, E11.3593, E11.3599, E11.36, E11.37X1, E11.37X2, E11.37X3, E11.37X9, E11.39, E11.40, E11.41, E11.42, E11.43, E11.44, E11.49, E11.51, E11.52, E11.59, E11.610, E11.618, E11.620, E11.621, E11.622, E11.628, E11.630, E11.638, E11.641, E11.649, E11.65, E11.69, E11.8, E11.9, E13.00, E13.01, E13.10, E13.11, E13.21, E13.22, E13.29, E13.311, E13.319, E13.321, E13.3211, E13.3212, E13.3213, E13.3219, E13.329, E13.3291, E13.3292, E13.3293, E13.3299, E13.331, E13.3311, E13.3312, E13.3313, E13.3319, E13.339, E13.3391, E13.3392, E13.3393, E13.3399, E13.341, E13.3411, E13.3412, E13.3413, E13.3419, E13.349, E13.3491, E13.3492, E13.3493, E13.3499, E13.351, E13.3511, E13.3512, E13.3513, E13.3519, E13.3521, E13.3522, E13.3523, E13.3529, E13.3531, E13.3532, E13.3533, E13.3539, E13.3541, E13.3542, E13.3543, E13.3549, E13.3551, E13.3552, E13.3553, E13.3559, E13.359, E13.36, E13.39, E13.40, E13.41, E13.42, E13.43, E13.44, E13.49, E13.51, E13.52, E13.59, E13.610, E13.618, E13.620, E13.621, E13.622, E13.628, E13.630, E13.638, E13.641, E13.649, E13.65, E13.69, E13.8, E13.9 | |
| **COPD** | J40, J41.0, J41.1, J41.8, J42, J43.0, J43.1, J43.2, J43.8, J43.9, J44.0, J44.1, J44.9, J47.0, J47.1, J47.9 | 490, 491.0, 491.1, 491.8, 491.9, 492.0, 492.8, 491.20, 491.21, 491.22, 494.0, 494.1, 496 |
| **Cancer** | C50.011, C50.012, C50.019, C50.021, C50.022, C50.029, C50.111, C50.112, C50.119, C50.121, C50.122, C50.129, C50.211, C50.212, C50.219, C50.221, C50.222, C50.229, C50.311, C50.312, C50.319, C50.321, C50.322, C50.329, C50.411, C50.412, C50.419, C50.421, C50.422, C50.429, C50.511, C50.512, C50.519, C50.521, C50.522, | 174.0, 174.1, 174.2, 174.3, 174.4, 174.5, 174.6, 174.8, 174.9, 175.0, 175.9, 233.0, V10.3, DX 153.0, 153.1, 153.2, 153.3, 153.4, 153.5, 153.6, 153.7, 153.8, 153.9,154.0,154.1, 230.3, 230.4, V10.05, V10.06, DX 185, 233.4, V10.46, DX 162.2, 162.3, 162.4, 162.5, 162.8, 162.9, 231.2, V10.11, DX 182.0, 233.2, V10.42 |

| | | |
|---|---|---|
| | C50.529, C50.611, C50.612, C50.619, C50.621, C50.622, C50.629, C50.811, C50.812, C50.819, C50.821, C50.822, C50.829, C50.911, C50.912, C50.919, C50.921, C50.922, C50.929, D05.00, D05.01, D05.02, D05.10, D05.11, D05.12, D05.80, D05.81, D05.82, D05.90, D05.91, D05.92, Z85.3, DX C18.0, C18.1, C18.2, C18.3, C18.4, C18.5, C18.6, C18.7, C18.8, C18.9, C19, C20, D01.0, D01.1, D01.2, Z85.038, Z85.040, Z85.048, DX C61, D07.5, Z85.46, DX C34.00, C34.01, C34.02, C34.10, C34.11, C34.12, C34.2, C34.30, C34.31, C34.32, C34.80, C34.81, C34.82, C34.90, C34.91, C34.92, D02.20, D02.21, D02.22, Z85.110, Z85.118, DX C54.1, C54.2, C54.3, C54.8, C54.9, D07.0, Z85.42 | |
| **Chronic kidney disease** | A18.11, A52.75, B52.0, C64.1, C64.2, C64.9, C68.9, D30.00, D30.01, D30.02, D41.00, D41.01, D41.02, D41.10, D41.11, D41.12, D41.20, D41.21, D41.22, D59.3, E08.21, E08.22, E08.29, E08.65, E09.21, E09.22, E09.29, E10.21, E10.22, E10.29, E10.65, E11.21, E11.22, E11.29, E11.65, E13.21, E13.22, E13.29, E74.8, I12.0, I12.9, I13.0, I13.10, I13.11, I13.2, I70.1, I72.2, K76.7, M10.30, M10.311, M10.312, M10.319, M10.321, M10.322, M10.329, M10.331, M10.332, M10.339, M10.341, M10.342, M10.349, M10.351, M10.352, M10.359, M10.361, M10.362, M10.369, M10.371, M10.372, M10.379, M10.38, M10.39, M32.14, M32.15, M35.04, N00.0, N00.1, N00.2, N00.3, N00.4, N00.5, N00.6, N00.7, N00.8, N00.9, N01.0, N01.1, N01.2, N01.3, N01.4, N01.5, N01.6, N01.7, N01.8, N01.9, N02.0, N02.1, N02.2, N02.3, N02.4, N02.5, N02.6, N02.7, N02.8, N02.9, N03.0, N03.1, N03.2, N03.3, N03.4, N03.5, N03.6, N03.7, N03.8, N03.9, N04.0, N04.1, N04.2, N04.3, N04.4, N04.5, N04.6, N04.7, N04.8, N04.9, N05.0, N05.1, N05.2, N05.3, N05.4, N05.5, N05.6, N05.7, N05.8, N05.9, N06.0, N06.1, N06.2, N06.3, N06.4, N06.5, N06.6, N06.7, N06.8, N06.9, N07.0, N07.1, N07.2, N07.3, N07.4, N07.5, N07.6, N07.7, N07.8, N07.9, N08, N13.1, N13.2, N13.30, N13.39, N14.0, N14.1, N14.2, N14.3, N14.4, N15.0, N15.8, N15.9, N16, N17.0, N17.1, N17.2, N17.8, N17.9, N18.1, N18.2, N18.3, N18.4, N18.5, N18.6, N18.9, N19, N25.0, N25.1, N25.81, N25.89, N25.9, N26.1, N26.9, Q61.02, Q61.11, Q61.19, Q61.2, Q61.3, Q61.4, Q61.5, Q61.8, Q62.0, Q62.2, Q62.10, Q62.11, Q62.12, Q62.31, Q62.32, Q62.39, R94.4 | 016.00, 016.01, 016.02, 016.03, 016.04, 016.05, 016.06, 095.4, 189.0, 189.9, 223.0, 236.91, 249.40, 249.41, 250.40, 250.41, 250.42, 250.43, 271.4, 274.10, 283.11, 403.01, 403.11, 403.91, 404.02, 404.03, 404.12, 404.13, 404.92, 404.93, 440.1, 442.1, 572.4, 580.0, 580.4, 580.81, 580.89, 580.9, 581.0, 581.1, 581.2, 581.3, 581.81, 581.89, 581.9, 582.0, 582.1, 582.2, 582.4, 582.81, 582.89, 582.9, 583.0, 583.1, 583.2, 583.4, 583.6, 583.7, 583.81, 583.89, 583.9, 584.5, 584.6, 584.7, 584.8, 584.9, 585.1, 585.2, 585.3, 585.4, 585.5, 585.6, 585.9, 586, 587, 588.0, 588.1, 588.81, 588.89, 588.9, 591, 753.12, 753.13, 753.14, 753.15, 753.16, 753.17, 753.19, 753.20, 753.21, 753.22, 753.23, 753.29, 794.4 |
| **Myocardial infarction** | I21.01, I21.02, I21.09, I21.11, I21.19, I21.21, I21.29, I21.3, I21.4, I21.9, I21.A1, I21.A9, I22.0, I22.1, I22.2, I22.8, I22.9 | 410.01, 410.11, 410.21, 410.31, 410.41, 410.51, 410.61, 410.71, 410.81, 410.91 |

| | | |
|---|---|---|
| **Organ transplant** | Z94.0, Z94.1, Z95.3, Z94.5, Z94.6, Z94.7, Z94.2, Z94.4, Z94.81, Z94.84, Z94.83, Z94.82, Z94.89, Z94.9 | V42.0, V42.1, V42.2, V42.3, V42.4, V42.5, V42.6, V42.7, V42.8, V42.81, V42.81, V42.82, V42.83, V42.84, V42.85, V42.86, V42.87, V42.88, V42.89, V42.9 |
| **Peripheral vascular disease** | I70.x, I71.x, I73.1, I73.8, I73.9, I77.1, I79.0, I79.2, K55.1, K55.8, K55.9, Z95.8, Z95.9 | 443.9, 441.x, 785.4, V43.4, 093.0, 437.3, 440.x, 441.x, 443.1, 443.2, 443.3, 443.4, 443.5, 443.6, 443.7, 443.8, 443.9, 447.1, 557.1, 557.9, V43.4 |
| **Cerebrovascular Disease** | G45.x, G46.x, H34.0, I60.x-I69.x | 362.34, 430.x, 431.x, 432.x, 433.x, 435.x, 436.x, 437.x, 438.x |
| **Renal disease** | I12.0, I13.1, N03.2, N03.3, N03.4, N03.5, N03.6, N03.7, N05.2, N05.3, N05.4, N05.5, N05.6, N05.7, N18.x, N19.x, N25.0, Z49.0, Z49.1, Z49.2, Z94.0, Z99.2 | 403.01, 403.11, 403.91, 404.02, 404.03, 404.12, 404.13, 404.92, 404.93, 582.x, 583.0, 583.1, 583.2, 583.3, 583.4, 583.5, 583.6, 583.7, 585.x, 586.x, 588.0, V42.0, V45.1, V56.x |
| **Asthma** | DX J45.20, J45.21, J45.22, J45.30, J45.31, J45.32, J45.40, J45.41, J45.42, J45.50, J45.51, J45.52, J45.901, J45.902, J45.909, J45.990, J45.991, J45.998 | |
